# Age-Related Hormones Changes and Its Impact on Health Status and Lifespan

**DOI:** 10.14336/AD.2022.1109

**Published:** 2023-06-01

**Authors:** Betina Biagetti, Manel Puig-Domingo

**Affiliations:** ^1^Endocrinology & Nutrition Service, Vall d’Hebron University Hospital and Vall d'Hebron Research Institute (VHIR), Department of Medicine, Autonomous University of Barcelona, Barcelona, Spain.; ^2^Endocrinology & Nutrition Service, Germans Trias Hospital and Research Institute, Badalona, Department of Medicine, Autonomous University of Barcelona, Badalona, Spain.

**Keywords:** older, elderly, diagnostic test, hormone, lifespan

## Abstract

The increase in life expectancy is accompanied with an increased consultation of age-related pathologies including endocrine disorders. Two main areas are focusing the attention of medical and social research in older population: the diagnosis and care of this heterogeneous population, and the interventional measures potentially useful to mitigate age-related functional declines and to increase health and quality of lifespan. Thus, better understanding the physiopathology of aging and establishing accurate diagnostic and personalized approaches are a priority and currently an unmet need of the medical community. The endocrine system plays a major role in survival and lifespan through regulating vital processes such as energy consumption and optimizing the stress response among others. The aim of this paper is to review the physiological evolution of the main hormonal functions in aging and its clinical translation to improve our approach to the aging patient.

## 1. Introduction

Aging can be defined as a state of progressive functional decline accompanied by an increase in mortality and an impairment of independent daily living activities. Older individuals, arbitrarily defined as those aged ≥65 years old, are a heterogeneous subset of people. Population censuses consider only chronological age for demo-graphic assessment, while biological age, which is determined certainly but not only by chronological age, is very much influenced by other factors that determine the final functional state.

Different categories of aging have been described according to specific criteria [1] such as successful or robust aging, pre-frailty, frail condition and finally disabled in pathological aging situations.

Due to the increasing life expectancy of western societies, aging is currently a hot topic either from social, economic or sanitary aspects. In particular, two areas are focusing the attention of medical and social research: the specificities for achieving a correct medical diagnosis and care of this heterogeneous population and the measures potentially useful to attenuate or prevent age-related functional declines, thus promoting an increase in health and quality of lifespan. Recently, a new field has been established within biological ageing research, namely 'geroscience', which is focused on health span extension [2].

With aging, the peripheral endocrine glands, and the central pituitary/ hypothalamic axis experience changes in its homeostasis. These changes could be related to (I) the patterns of hormone secretion capacity; (II) the receptor response to hormones and (III) the peripheral metabolization of hormone products.

Increase in life expectancy is accompanied by an increased consultation for age-related pathologies including endocrine disorders. In this context, there is growing awareness that natural changes of endocrine physiology and physiopathology occurring with aging may require specific diagnostic cut-offs and their validation in the older individuals [3]. Thus, better understanding the hormone physiology and pathophysiology evolution with aging is a priority and currently an unmet need.

The change in body composition, loss of muscle strength, anorexia observed in older adults, decline in mental and mood conditions and enhanced tumorigenesis may be due, or at least influenced by changes of different magnitudes of the endocrine function contributing in a variable extent to this specific phenotype.

Indeed, the endocrine system plays a major role in survival and lifespan. From an evolutionary perspective, the endocrine system is responsible for the metabolic adaptation of the species. According to the “feast and famine cycle” hypothesis [4], ancestral adjustments to survival could be responsible for the nowadays pandemics of insulin resistance, obesity and cardiovascular risk, as a consequence of the bias selection of the best saver individuals [5]. Thus, the endocrine system and its deep interplay with all body organs and systems intervene in the conservation of the species, regulating a vital process such as energy consumption and ensuring the species maintenance through optimization of the stress response and the reproductive process.

The aim of this paper is to review the physiological evolution of the main hormones in aging and its clinical translation to improve our approach to aging patients, as well as gaining deeper insight into aging hormones homeostasis and the -sometimes- uncertain interpretation of hormone circulating concentrations and pattern in this life stage.

The specific aims of this review are listed below:

To describe the evolution of the main hormones in aging

To interpret the meaning of hormonal changes in aging

## 2. Methods

This review was conducted following SANRA scale[6]. We have carried out the search in PubMed by two steps. In the first step we limited the search to articles published in English in the last 20 years with the following terms: ("aging" [Title] AND "hormones" [Title]) AND ((2000:2022/7 [pdat]) AND (English [Filter])): This strategy found 126 articles.

The second step search was a more specific approach, for each item, also limited to English language but restricted to the last ten years, the search terms are listed below:

- ("aging" [Title] AND ("menopause" [Title] OR "hypogonadism" [Title])) AND ((2010:2022/7 [pdat]) AND (English [Filter])): this search returns 112 articles
- ("aging" [Title] AND "growth hormone" [Title]) AND ((2010:2022/7 [pdat]) AND (English [Filter])): 53 articles
- ("aging" [Title] AND "thyroid" [Title]) AND ((2000:2022/7[pdat]) AND (English [Filter])): this search returns 72 articles
- ("aging"[Title] AND "prolactin"[Title]) AND ((2010:2022/7[pdat]) AND (English [Filter])): 5 articles
- ("aging" [Title] AND "adrenal" [Title]) AND ((2010:2022/7 [pdat]) AND (English [Filter])): this search returns 20 articles

These searches resulted in a total of 368 articles, of these, 40 were excluded because they did not have available abstract and 97 were duplicated. After abstract review we eliminate 37 older papers authored by the same research group or focused on similar topics. Finally, 194 papers were reviewed along with some relevant references published before 2000 cited in those articles.

The information of these articles was synthesized to draw a final conclusion.

## 3. Hormonal changes and aging

After birth, the pituitary gland quickly adapts hormone balances to the new extrauterine life, first in early-post-natal period thereafter in puberty, pregnancy, lactation, but also in stress situations and illnesses. In contrast, this adaptive capacity may be compromised during aging concurrent with pituitary and peripheral endocrine glands functional decline [7].

The hormone decline in older people is in most cases referred as “pause” (i.e. menopause, andropause, adrenopause, and somatopause) [8]. In the next section we will review the hormone changes in this life stage.

## 4. Result

### Adenohypophysis

#### 4.1. Gonadotropic axis and physiology of aging

##### Physiology of aging in women

Orthochronic spontaneous menopause is an example of naturally programmed end of a physiological process, in this case the reproductive function. Menopause triggers significant clinical consequences [9] associated with an abrupt loss of estrogen and progesterone production in women due to the cessation of ovarian function. It is characterized biochemically by serum concentrations of Follicule Stimulating hormone (FSH) and Luteinizing hormone (LH) >25 mIU/mL along with estradiol levels < 50 pmol/l and amenorrhea for 12 consecutive months [10]. There is some evidence of increasing age at menopause over time [11], but in most ethnic groups menses cessation occurs in a very narrow age frame between 50 and 51 years old [11-13]. Follicular atresia and not the absolute number of oocytes is the main cause of the loss ovarian cyclicity at least in mice [14],although, menopause is reached when follicles depletion reaches approximately 1000 (from a peak of 5 million follicles in mid-gestation and 2 million at birth) [11]. The follicular reduction entitled a declining of serum concentrations of granulosa cell-secreted anti-Müllerian hormone (a marker of ovarian reserve), and inhibin B (a marker of ovarian activity) produced predominantly in developing antral follicles during the follicular phase of the menstrual cycle [15, 16].

The time of menopause onset is chronologically age-dependent, but other factors influence this event like genetics, immune factors, menarche onset, lactation, and parity [17] although the exact molecular mechanisms that finally determine the decline in oocyte/follicle quality and function are still to be determined.

Circadian clock function, like most physiological processes, is involved in the events that determine reproductive aging. The involvement of the suprachiasmatic nucleus (SCN) and the activity of vasopressinergic neurons in maintaining the rhythmicity of the female reproductive system depend on the mRNA transcription-translation feedback loops [18]. Expression of clock genes, *Per2*, *Bmal1*, and *Rev-erbα*, among others, in the hypothalamus-pituitary-gonadal axis and the vasopressinergic neurons activity in the SCN and kisspeptin neurons in the arcuate nucleus (ARC) play important roles in the gonadotropic function cessation/decline of aging. Changes in the temporal synchrony of the clock system in the hypothalamus-pituitary-gonadal axis during the period prior to the cessation of ovulatory cycles have been identified [18]. The desynchronization between the central and peripheral circadian clocks contributes to the irregularity of reproductive events, indicating that the feedback loops of clock genes on the hypothalamic-pituitary-gonadal axis modulates the spontaneous transition from regular to irregular cycle and to acyclicity in female, at least in experimental models.

In contrast, the andropause is a gradual and heterogeneous decline in testosterone that begins at around 30 to 40 years of age and persists until death [19].

**Figure 1.**
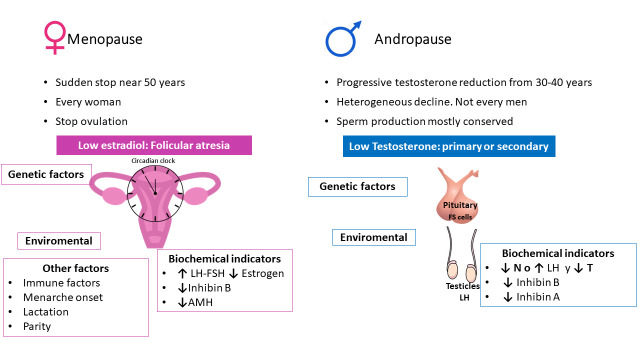
**Menopause *vs* Andropause.** The differences between menopause and andropause are outlined FS: Folliculostellate cells, LH: Luteinizing hormone; AMH: Anti-Müllerian hormone.

##### Physiology of aging in men

Classically, similar than in women, the testosterone decline in men has been related with an impaired testicular testosterone secretion, namely, gonadal primary decline theory[20], in which impaired testosterone feedback at hypothalamic and pituitary level unleashes LH secretion during the aging process. Nevertheless, the changes that take place in the testicles during aging run along with an increase in Folliculostellate cells (FS), a non-hormonal cell subtype which comprises 5-10% of the anterior pituitary cells population and influences the function of the neighboring hormonal cells in a paracrine manner [21]. During the aging process, the density and size of the FS cells increase in parallel with a hypertrophy of LH cells that could be related with their functional decline in older men. The strong correlation between FS cells and LH cells morphometric aging changes might suggest an increasing functional interaction between these two groups of cells that could be of causative nature. These results, opposite to the classical peripheral gonadal decline, point out to primary pituitary changes and paracrine signals as a feasible start point of men’s gonadotroph axis aging.

These data indicate that the mechanisms involved in gonadopause have not yet been fully clarified, and that although primary impaired gonadal function by aging has been traditionally considered as the principal driver, primary pituitary changes could also be of most relevance, opening a new perspective in hypothalamic-pituitary-gonadal aging process, at least in men. Moreover, the abrupt cessation of reproductive function in women, from an evolutionary point of view, may confer protection of the species as a pregnancy over 50 years old could threaten the life of the pregnant woman hindering foetus survival. On the other hand, male’s reproductive function could continue as a species survival mechanism (Fig. 1).

##### Experimental evidence of gonadotropic axis action on lifespan

Mouse models’ experiments show that gonadotropic axis has an important role in longevity [22]. With aging there is an attenuation of hypothalamic GnRH gene expression. Zhang G. et al. [22], using GT1-7 cells, a cell line of GnRH neurons, demonstrated that IKKβ/NFκB activation (an aging pathway which is chronically activated in aging), can strongly inhibit GnRH gene transcription, thus providing an explanation for the phenomenon of aging-related GnRH decline. On the other hand, they demonstrated that GnRH administration significantly reversed aging-impaired neurogenesis in the hypothalamus, which led to amelioration of various aging effects, including skin atrophy, muscle weakness, and bone loss (Table 1).

**Table 1:** Summary of the impact of hormone changes or hormonal administration in lifespan in experimental animal models.

Gonadotropic axis	
• Inhibition of GnRH by NF-κB initiates aging. Mice model and GT1-7 cells lines [22]	
• GnRH therapy can greatly amend aging disorder. Mice and GT1-7 cells lines [22]	
**Growth hormone and Insulin like growth factor -1 axis**	
**GH deficient transgenic mouse**	Increase LE 9-68% [56]
**GH resistant transgenic mouse**	Increase LE 0-55% (some mutations only extend LE in female) [56]
**Reduced IGF-1**	Increase LE 0-20% (LID mutation decreases LE in male and iLID do not change LE [56]
**Transgenic mouse**	Cardiac aging was cancelled by IGF-1 deficiency [56]
• GH treatment in old mice was able to show beneficial effects on different organs and metabolic functions [64]	
**Klotho**	
• A defect in Klotho gene expression is an accelerated aging model in mice [60] • Overexpression of Klotho in mice extends life span [62]	
**Thyrotropic axis (conflicting data)**	
• Experimentally induced hypothyroidism prolonged life in rats [84]. • The administration of TRH to old mice showed aging-delaying and aging-reversing effects [85] • DNA damage could be behind thyroid state and a tissue-specific regulation of deiodinase activities, could be a protective metabolic response in aging [86].	
**Prolactin axis**	
• Ames and Snell transgenic model entail hypopituitary dwarf mice with GH, TSH and prolactin deficiencies, having 40-60% LE [55] • PRL mutants’ mice (PRLR^-/-^) or doble mutants (D2R^-/-^, PRLR^-/-^) mice, do not extend life expectancy [103].	
**Corticotropic axis**	
• Not useful for LE models	

LE, life expectancy; LID, liver IGF-I ablated; iLID, inducible liver-specific

#### Gonadotropic axis and aging pathology

##### Aging pathology in women

Menopause can occur earlier than usual; defined as the permanent cessation of menses before the age of 45 years old, or much earlier, called in this case premature ovarian failure when the cessation of menses occurs at the age < 40 years old [23]. In addition to the obvious reduction in fertility, premature menopause entails metabolic, cardiovascular, osteoarticular, genitourinary, sexual and mood disturbances [24].

On the other side, late-onset menopause is considered when a woman is 55 years or older and still has not begun menopause. These women have an increased exposure to estrogens and have an increased risk of hormone-dependent cancers [25-27]. Both, early and late onset menopause show a U-shaped relationship with non- procedure-related venous thromboembolism independently of hormonal therapy [28]

##### Aging pathology in men

Differently that what happens in women, men have a modest and heterogeneous age-related decline in circulating testosterone levels with high interindividual variability [19], which is paralleled by deterioration of sperm quality [29] and in some cases results in the development of symptomatic hypogonadism, which has been called late onset hypogonadism [30]. The definition of this latter has been a matter of discussion but in a substantial part of individuals affected, a clear relationship with obesity is present [31].

#### Treatment of hypogonadism in older people

##### Hormonal replacement in women

The publication of the Women's Health Initiative (WHI) results in 2002 [32], strongly decreased the use of hormone replacement in menopausal woman from 25-30% to 3-4% of peri/postmenopausal women [33, 34]. The new evidence obtained in the last decade demonstrating the safety of hormonal replacement therapy in menopausal women with vasomotor and urogenital symptoms, as well as the prevention of osteoporosis has led to updating the recommendations [35]. However, the duration of hormone replacement exposure is relevant. The relative risk of breast cancer significantly increases when it is started in women aged 50 years or older (relative risk, 1.35) and its extends for more than five years [36, 37], but it is not increased when hormonal replacement therapy is given for premature menopause and is stopped at the age of fifty. Thus, oestrogens and progestogens should be used at the lowest dosages and for the shortest durations necessary to achieve the physiologic goals of health protection as well as symptoms relief.

##### Hormonal replacement in men

In men, while all guidelines agree that a combination of symptoms of testosterone deficiency and low serum testosterone levels establish late onset hypogonadism and are prerequisites for testosterone substitution, there is still no agreement on the specific threshold levels at which testosterone therapy should be given [38, 39]

### 4.2 Somatotropic axis

#### Somatotropic axis physiology in aging

Somatopause is the term used to define the decline in pulsatile secretion of growth hormone (GH) and its corresponding decremental effect on circulating insulin-like growth factor 1 (IGF-1) that occurs with age. It is associated with changes in body composition and physical and psychological function that paddles those seen in younger adult patients with growth hormone deficiency, including reductions in lean body mass and muscle strength and an increase in body fat, particularly in the visceral compartment [40]. Moreover, skin texture, sleep pattern and coagulation changes have also been related with GH impairment [41].

After the third decade of life, there is a progressive decline of GH secretion. This process is characterized by a loss of day-night GH rhythm that may, in part, be related with the aging-associated loss of nocturnal sleep. Age-related GH decline is a complex process and other mechanisms as decreased secretion of GHRH and an enhancement of the inhibitory effect of somatostatin or pituitary GHRH resistance are a matter of debate. Supporting this, some studies have shown aged-related decreases in hypothalamic GHRH content and an increase of hypothalamic somatostatin activity in rats [42] and monkeys [43].

Beside internal regulators, environmental agents and disruptors may also impact negatively the somatotropic function. Thus, mercury deposition in human pituitary glands at different ages has been found and a high mercury content associated with increasing age, suggesting that mercury toxicity could be one of the environmental factors contributing to the decline in growth hormone levels in aging [44].

**Figure 2.**
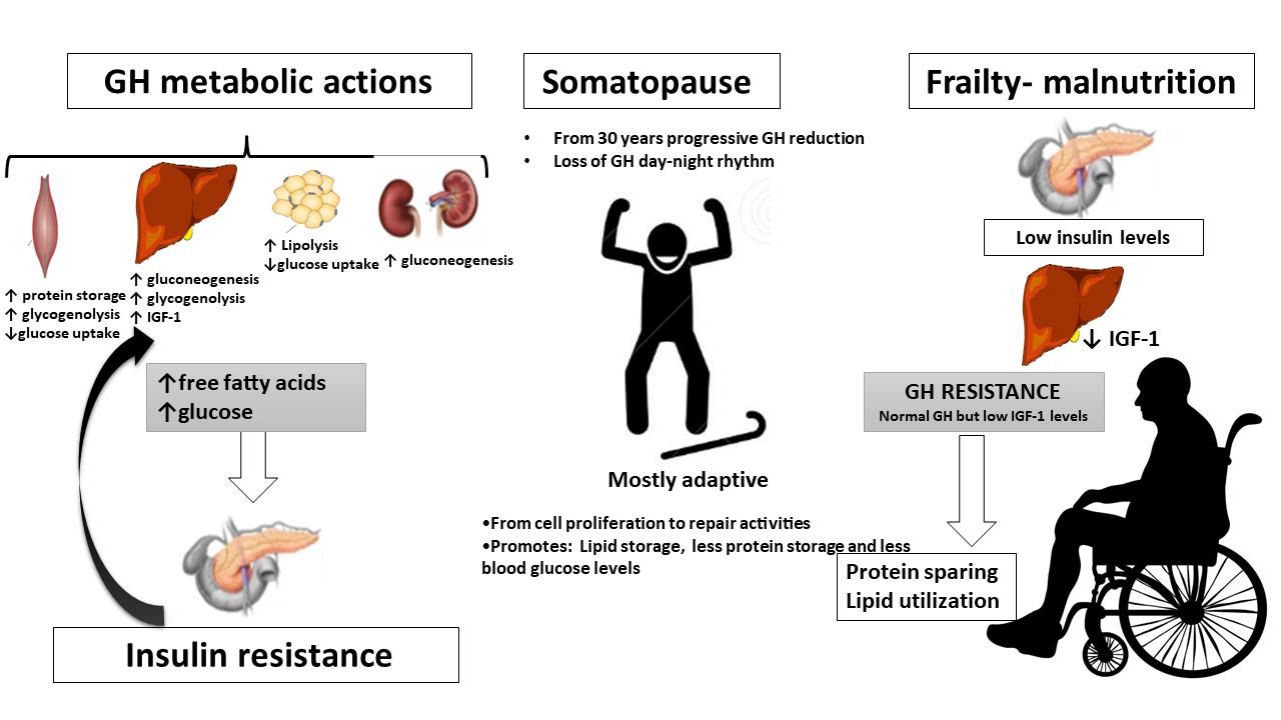
**Growth hormone metabolic actions in healthy aging and frailty.** The figure shows the metabolic actions of growth hormone its consequences in somatopause and GH axis alteration with malnutrition.

On the other hand, aging decline in IGF-1 production seems to be a direct result of the GH decrease. In contrast to other hormonal systems, such as insulin in which a progressive insulin resistance is observed while aging, there is no evidence indicating that GH resistance develops in older adults. Actually, in patients with GH deficiency, the dose of GH necessary to maintain normal IGF-1 concentrations are lower in older subjects [45]. However, insulin levels change dramatically in older population depending on nutritional state, which directly impacts GH and IGF-1 circulating concentrations [46, 47]. Therefore, GH and IGF-1 levels should be carefully evaluated taking into account the nutritional state, as low insulin levels present in malnutrition make the liver resistant to GH [48, 49] contributing to reduce even more circulating IGF-1 levels. In addition, GH liver resistance could be an adaptive response to undernutrition to maintain euglycemia, preserving proteins, switching to lipolysis as primary source of energy (Fig. 2).

Likewise, different genetic factors, such as IGF-1 polymorphisms may modulate IGF-1 production through the whole lifespan and impact quality of life and longevity [50-52]

Endocrine and metabolic adaptation observed in older population during caloric restriction may be a physiological strategy for extending lifespan through a slower cell growing and metabolic rate, a better physiologic reserve capacity, a shift of cellular metabolism from cell proliferation to repair activities and a decrease in accumulation of senescent cells [49]. From a teleological point of view, these facts seem to indicate that somatopause is among other, an adaptive mechanism to better aging.

#### Experimental evidence of somatotropic axis and lifespan

One of the most undebatable and consistently reproduced experimental finding indicates that down regulation of GH signaling in animal models, such as in the caenorhabditis elegans[53] dwarf mice models[54-56],and models of IGF-1 deficiency[56-59] leads to remarkably long-lived species, both in mean and maximum lifespan, exhibiting multiple characteristics that suggest delayed aging (Table 1).

Likewise, klotho, a trans-membrane enzyme with a soluble circulating isoform highly expressed in the brain, the kidney, the parathyroid and pituitary glands, shows important anti-aging activity. Mice that do not express klotho die prematurely with multiple symptoms of aging, several of which are also characteristic of decreased GH/IGF-1 axis activity [60, 61] and it overexpression in mice extends life span [62] Several lines of evidence suggest an association between klotho circulating levels and the activity of the GH/IGF-1 axis. Thus, klotho is a player in the intricate regulation of the GH/IGF-1 axis that could participate in the modulation of aging phenotype and the length of lifespan. Although some genetic variants have been described as the drivers of increased longevity in mice, the translation to humans has not confirmed such findings, given replication studies performed with the UK Biobank cohort did not support the role of klotho as a longevity factor in humans [63].

In contrast to the down regulation of GH/IGF-1 axis and extended lifespan, GH treatment in old animals is able to show beneficial effects on different organs and metabolic functions [64], thus, reinforcing the concept that GH/IGF-1 axis action on aging and longevity is complex. Conversely, GH deficiency [65] or insensitivity [66] in humans do not extend human longevity although it confers a protective effect against cancer and diabetes. However, pathological elevation of GH levels in acromegaly reduces health span and longevity. Moreover, aging is associated with a continuous circulating GH reduction evidenced in several epidemiological studies in humans [67, 68]. Therefore, the reduction of GH levels in the older population is very characteristic but its meaning is controversial.

#### Somatotropic axis pathology and treatment in aging

According to an ESE audit [69], 5% of patients with adult GH deficiency (AGHD) is older than 65 years at diagnosis. Symptoms and signs of AGHD are non-specific, thus consensus clinical practice guidelines suggest to perform diagnostic testing only in adults with a reasonable probability of having the deficit (e.g. history of hypothalamic/pituitary disorders, surgery and/or cranial irradiation to this region in the brain), and with the aim to offer treatment when the diagnosis is confirmed [70-72]. However, the clinical benefit of initiating GH therapy in older patients is a matter of debate [73]. Moreover, cut-off values could be different, as reported by Colao et al. [74], which found that individuals older than 65 yr have lower cut-off points than do middle-aged adults. Further studies are still needed to validate different cut-points for distinct patient’s age populations. Likewise, there is a high variability regarding GH treatment reimbursement among countries, in part related to the lack of perception of the benefits of GH replacement therapy by some healthcare professionals and administrators, which is even more pronounced in those patients over 65 years. Therefore, there is no agreement regarding the justification of evaluating GH deficiency in patients over 65 years of age. In addition, there is also no agreement regarding the age at which treatment with GH would no longer provide benefits or the risks would increase, and therefore treatment should be discontinued or not after individual evaluation of every case.

**Figure 3.**
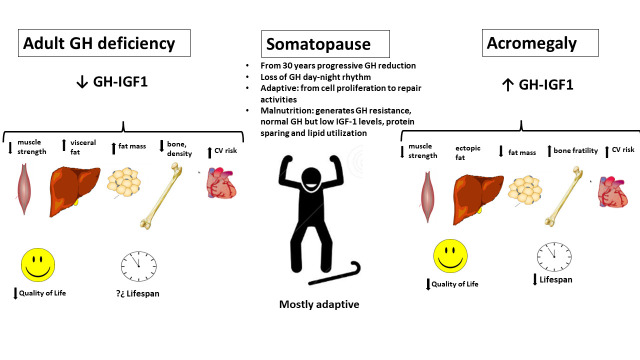
**Growth hormone axis physiology and pathology spectrum.** The figure shows the spectrum of GH axis physiopathology: GH deficiency, Somatopause and Acromegaly.

As to GH excess, acromegaly diagnosis is growing in older patients [75, 76], but, there are no age-adapted diagnosis or protocols [77-80]. Our group found that GH< 6ng/ml at diagnosis and female gender, but not age per se, were associated with greater chance of response to somatostatin receptor ligands (SRL) treatment, thus confirming the heterogeneous nature of the disease, which also applies to patients aged 65 years or older [81] (Fig. 3).

### 4.3 Thyrotropic axis

#### Thyrotropic axis physiology of aging

Thyroid function change with aging towards a circulating hormonal profile showing higher TSH and lower free T3 and free T4 concentrations in humans [82]. Thyroxine secretion is slightly reduced in aged individuals as it is the capacity to metabolize T4 by deiodination in certain tissues of healthy aged individuals, albeit with the net result of normal circulatingT4 levels [83].

#### Experimental evidence of thyrotropic axis and lifespan

There is conflicting data regarding the effect of thyroid hormones in life expectancy. Experimentally induced hypothyroidism prolonged life in rodents[84, 85] and the administration of TRH to old mice showed aging-delaying and aging-reversing effects[86]. More recently, Visser et al [87] showed that DNA damage was the underlying mechanism of changes related to aging in thyroid state and a tissue-specific regulation of deiodinase activities, could be a protective response in aging.

#### Thyrotropic axis pathology and treatment in aging

Rakov H, et al. [88] studied the effect of treatment of young and old mice at baseline and after an acute T4 challenge. Thyroid hormones levels were lower in old versus young mice. After T4 injection, the conversion of T4 to T3 was decreased in old mice while the thyroid hormones transport in liver and heart was not affected. Organ-specific response was augmented in old mice in liver but not heart, indicating an age- and tissue-specific different sensitivity to T4. This suggests a reduced activity of the aged thyroid that could be responsible for the systemic low thyroid hormone state in old mice. Furthermore, divergent tissue responses in liver and heart after T4 treatment could justify a narrower window for T4 substitution in the older adults to avoid overtreatment.

In humans, the prevalence of thyroid dysfunction increases with age, but it is associated with an attenuated and oligosymptomatic clinical presentation of either hyper- and hypothyroidism; indeed, epidemiological studies have associated subclinical hypothyroidism with a reduced risk of all-cause mortality [89-91] while, conversely, subclinical hyperthyroidism has been related to an excess of all-cause mortality and in particular cardiac-related death [92]. Actually, decreased thyroid function in older adults is suggested to contribute to an increased lifespan [89, 93]; moreover, mild subclinical hypothyroidism in older age, besides being a protector of heart death, does not show an impairment in physical function in terms of muscle strength [94] and could be an adaptive protective mechanism [95]. This has raised the discussion regarding to what extent TSH upper limits of normality should be reformulated in older adults. Consistent with that, experimental thyroid gland ablation in different animal models [96] generally result in extended lifespan compared with control animals with normal thyroid activity. These results evidence that thyroid hormones play an important role in the duration of life and specifically a mild hypothyroid state in humans could be positively related with longevity and should not be misinterpreted.

Regarding treating older individuals with subclinical hypothyroidism, generally, it is accepted that treatment is not necessary unless TSH exceeds 10 mIU/L. In double-blinded randomized controlled trials, treatment did not improve symptoms or cognitive function if the TSH was lower than 10 mIU/L [97] and normalization of TSH with levothyroxine was associated with no difference in atherosclerosis in older persons [98] and even could be harmful[99].

Conversely, subclinical hyperthyroidism is associated with an increased risk of coronary heart disease mortality, incident atrial fibrillation, heart failure, fractures and excess mortality in patients with serum TSH levels <0.1 mIU/L [92]. Therefore, treatment is indicated in all older people to potentially avoid these complications and the risk of progression to overt hyperthyroidism. In the case of TSH levels 0.1-0.39 mIU/L treatment could be also recommend due to an increased risk of atrial fibrillation, especially in the presence of symptoms and/or underlying risk factors or other co-morbidities [92].

To sum up, thyroid hormone axis activity declines with age, as reflected by a slightly increase in TSH and a decrease in T3 concentrations. These changes seem to be adaptative and as a consequence beneficials. On the contrary, subclinical hyperthyroidism could be deleterious. Therefore, the absence of age-specific hormone reference ranges should be promptly solved to avoid misdiagnosis in older people.

### 4.4 Prolactin axis

#### Prolactin axis physiology

The best known function of prolactin (PRL) is the growth and activation of the mammary gland for lactation while its exact role in the male is poorly understood [100]. Less well known functions of prolactin are related with metabolism [101], the immune system [102] and the regulation of adult stem/progenitor cells [103].

#### Experimental evidence of prolactin axis and lifespan

As we commented above, some rodent models in which GH/IGF-1 axis is attenuated show increased life expectancy. The Ames and Snell models, entail hypopituitary dwarf mice with GH, TSH and prolactin deficiencies, having 40-60% increases in life expectancy [56] (Table 1). Nevertheless, specifically PRL receptor mutants mice (PRLR^-/-^) or double mutants (D2R^-/-^, PRLR^-/-^) mice which are used to bypass the short loop feedback dopamine/PRL in pituitary, do not show extended life expectancy [104].

#### Prolactin axis pathology and treatment in older adults

While prolactin overproduction could lead to galactorrhea and has an inhibitory effect on the release of gonadotropin-releasing hormone that results in functional infertility, menstrual cycle disturbances in young women and decreased libido and spermatogenesis in men, the hypo-prolactinemic situation does not show any striking clinical picture apart of the impossibility to lactate during the puerperium. Nevertheless, in the EMAS study [105], a prospective, observational cohort of community-dwelling men aged 40-79 years old, it was found that low PRL levels were related to several dysfunctions at the metabolic, psychological, and sexual domains. Likewise, in older-old women (aged 100-115 years), serum PRL levels have been found significantly higher than those in young subjects, which has been related with dopaminergic/somatostatinergic aging changes [67]. A decrease in hypothalamic dopaminergic tone explaining this PRL increase has been reported in some animal aging studies [106, 107].

All these changes seem to be also of adaptive nature and should not be considered of pathologic significance; thus, prolactin levels in advanced healthy ages tend to be in the upper normal range or slightly higher than in young individuals. Therefore, treatment of spurious elevations of prolactin should be avoided ever, but even more in older people.

### 4.5 Corticotropic axis

#### Corticotropic axis physiology in older adults

Cortisol is one if not the principal key hormone in the stress response, together with vasopressin. Physiological adaptation of stress requires adrenal activation. However, chronic hypothalamic-pituitary-adrenal axis increased release of glucocorticoids induces deleterious effects at every tissue level with particular relevance to the brain [108]. Dysregulation of adrenal axis has been also reported in aging [109]. This includes an enhanced daily cortisol secretion, attenuated wake-evening slopes, a more pronounced cortisol awakening response [110], as well as a decline in adrenal androgen levels, mainly dehydroepiandrosterone (DHEA) [111]: this latter being postulated as a tissue protector in contraposition to cortisol [112]. Moreover, a U-shaped pattern across the life span in urinary free cortisol levels has recently been reported [113] although there is not always a correlation between plasma ACTH and corticosteroids [114] or gender [115]. Aging has also been associated with an increased expression of 11βHSD1 both in the brain and peripheral tissues at least in mice [116]. Such changes could expose tissues to elevated levels of glucocorticoids and contribute to deleterious aging process.

Higher urinary free cortisol concentrations have been associated with Alzheimer’s disease and in general, to a decline in cognitive function [117]. Moreover, basal levels of glucocorticoids, loss of circadian rhythm, increased cortisol/DHEA ratio [118] and changes in cortisol 30 minutes after awakening [119] have also been related with greater cognitive decline at a given age. A prospective study [119] with 625 dementia-free participants aged ≥65 years showed that at 5 years of follow-up, only longitudinal changes in the cortisol 30 minutes after waking remained positively associated with cognitive decline after controlling for potential confounders. In addition, a serum cortisol/DHEA ratio ≥0.2 has also been associated with sarcopenia in patients aged ≥65 years with type 2 diabetes [120].

The effects of aging on CRH, or how CRH influences the course of aging is a controversial issue. Studies have reported increased, unchanged, or reduced hypothalamic CRH release and expression during aging [121].

Regarding stress response, a study comparing postmenopausal women (age range 55-68 years) with 30 healthy young women (20-35-yr old), showed that maximal DHEA response to 250 μg of ACTH was significantly lower in the later, while cortisol output was slightly, albeit significantly increased in older women [122]. These results have not been replicated by other groups [123], thus the question is still open.

Overall, chronically elevated glucocorticoid levels in older individuals seems to have at least two main consequences, first a deleterious metabolic and direct organ effect in several tissues as brain and muscles, and second, a hyper-response to acute stress, reflecting an altered regulation of this protective mechanism.

#### Hypothalamus and Neurohypophysis

The hypothalamus concentrates the control of almost all homeostatic functions including circadian rhythms, body temperature, hunger, thirst, attachment behaviours, sleep, and fatigue/wakefulness [124], as well as hormonal production through its input to the pituitary function. As expected, a growing evidence confirms the relationship of aging to changes at the hypothalamic level [22, 125, 126].

One of the most relevant changes that occurs during aging is the modification of energy homeostasis, which may be related to an increasing incidence of metabolic syndrome. A second very relevant event observed in aging is the very frequent loss of appetite that has been called as the “aging anorexia syndrome” [127]. The determinants of both of these clinical situations associated to aging are multiple and heterogeneous, and far for being unravelled [46]. The underlying cellular mechanisms for the hypothalamus age-related aging progression comprises dysregulation of nutrient sensing, altered intercellular communication, stem cell exhaustion, loss of proteostasis, and different epigenetic alterations [128].

Finally, older people have impaired thirst and less capacity to cope with osmotic challenges such as dehydration. Dehydration-induced expression of genes in the supraoptic nucleus is attenuated in aged rats [129]. Tau protein deposition in the posterior pituitary has been implicated as a potential etiologic factor of age-related neuropituitary dysfunction [130].

## 5. Conclusions: Impact of ageing in endocrine clinical practice

Aging people represent a growing proportion of patients in our clinical practice: according to World Population Prospects 2019 [131], by 2050, 1 in 6 people in the world will be over the age of 65, while in 2019 it was estimated to be 1 in 11. Therefore, age-related pathology including endocrine disorders will become more prevalent in our clinics, and it is not expected to slow down in the next decades.

Older people are a heterogeneous population. From a medical point of view, when we face an older patient, we have two main challenges: (I) to interpret the diagnostic tests correctly and (II) to provide the best possible and personalized treatment. In this regard, it is well known that clinical trials under-enroll old subjects, thus, the diagnosis and treatment are applied following what it is recommended to the general much young population, which in general cannot be extrapolated to aged people.

In the last decade a considerable number of publications have tried to overcome this lack of information and have focused in enhancing a better characterization [132] of certain endocrine diseases [133-136], and their treatment options [137-139] in relation to age-related changes [140]. Very prevalent disorders at young-mid ages show an important increase in their prevalence with aging; of particular note for general physicians, geriatricians and also endocrinologists are type 2 diabetes, with a prevalence >25% (www.diabetes.org/resources/statistics/statistics-about-diabete), obesity and metabolic syndrome [141] which raises up to 50% and hypothyroidism which is present in about 5% of women older than 60 years [142].

**Figure 4.**
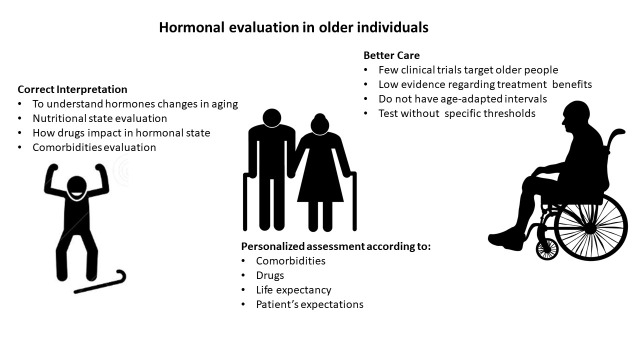
**Hormonal evaluation in older individuals.** The figure shows the complexity facing elder patients care, due to misinterpretations, few evidence-based medicine and the need of an age-personalized approach

It is important to stress that, the mutual impact of frailty in the development of endocrine disorders and vice-versa is a major issue which is sometimes even more challenging at the time of accurately interpret the results of diagnostic biochemical and hormonal tests and to improve clinical decision processes [143]. Chronological age is not the best marker to characterize older population. Patients with frailty could have increased length of stay and hospital work load i.e. when undergoing pituitary surgery [144]; moreover, the standard care could be self-defeating i.e., when dealing with intensive glucose-lowering and blood pressure-lowering treatments [145]. Basal hormones evaluation in persons 65 years and older requires differentiating the physiologic adaptive changes from those leading to an increased risk of disease (Table 2). In the older population, it is important to recognize the hormonal patterns evolution by the own age, gender, the nutritional state, concurrent drugs and associated comorbidities Figure 4.

**Table 2:** Usual basal hormones’ changes in older population and malnutrition impact.

Axis	Gender		Malnutrition
Gonadotropin	Male LH-FSH ↔/↓ T ↔/↓	Female LH-FSH ↑↑ E↓↓	LH-FSH ↓ T and E ↓
Somatotropin	GH ↓ IGF-1 ↓		GH ↑ IGF-1 ↓
Thyrotropin	TSH ↑ FT4 ↔ FT3 ↔ or ↓		TSH ↓ rT3↑
Prolactin	PRL↔ or slightly elevated		__
Corticotropin	• ↓ Negative feedback • ↑ awakening response •↑ daily cortisol secretion •↓wake-evening slopes • Irregular cortisol pattern •↓DHEA		↑CRH, ACTH, and cortisol;

The evaluation of basal hormones in older individuals should be assessed according to gender, and nutritional state. LH: Luteinizing hormone, FSH: follicular stimulating hormone, T: testosterone, E: estradiol, GH: growth hormone, IGF-1: insulin-like growth factor, IGFBP-3: IGF-binding protein 3, TSH: thyrotropin FT4:4- free thyroxine, FT3: 3- free thyroxine, ACTH: adrenocorticotropin, C: cortisol. PRL: prolactin.

Regarding dynamic test age-related cut-offs, this issue remains to be validated in persons 65 years and older and thus, further studies are needed to establish what specific test and cut-offs have to be applied in those cases which have not been performed, that are the majority. In relation to longevity, GH/IGF-1 axis is the most studied in rodents, although increased life expectancy has been shown in mice models of GH deficiency, GH resistance or low IGF-1 levels, the treatment of such deficits in older mice has also shown improvements in longevity thus, highlighting the differences of hormonal modification related to age in different species.

In human beings most of the changes observed in very older people or long-lived populations seem to support the idea that most of the hormonal changes are adaptive; moreover, the hormonal reversal to young subject’s values of these changes did not show benefits and in some cases may even be harmful.

In conclusion, aging is a complex process, hormone levels interpretation in this stage of life is challenging and requires deep knowledge of the natural physiology of aging to avoid misdiagnosis, but also a comprehensive functional and nutritional assessment of the patient and its social environment. Moreover, we are far from having personalized protocols for this heterogeneous population. We urgently need more studies focused in this population to give the best possible care.
